# Adult-Onset Still’s Disease-like Syndrome following COVID-19 Vaccination: A Case Report and Review of the Literature

**DOI:** 10.3390/vaccines10071022

**Published:** 2022-06-26

**Authors:** Poramed Winichakoon, Wanitcha Chanloung, Teerapat Nantsupawat, Worawit Louthrenoo

**Affiliations:** 1 Division of Infectious Diseases and Tropical Medicine, Department of Internal Medicine, Faculty of Medicine, Chiang Mai University, Chiang Mai 50200, Thailand; poramed.wi@cmu.ac.th; 2 Division of Rheumatology, Department of Internal Medicine, Faculty of Medicine, Chiang Mai University, Chiang Mai 50200, Thailand; wanitcha.ch@cmu.ac.th; 3 Division of Cardiology, Department of Internal Medicine, Faculty of Medicine, Chiang Mai University, Chiang Mai 50200, Thailand; teerapat.nant@cmu.ac.th

**Keywords:** adult-onset Still’s disease, autoinflammatory disease, vaccine, COVID-19, SARS-CoV-2

## Abstract

Adult-onset Still’s disease (AOSD)-like syndrome has rarely been reported as a complication of COVID-19 vaccination. This study reports a 31-year-old female patient who presented with fever, myalgia, arthralgia, pleuropericarditis, leukocytosis, and transaminitis following ChAdOx1 vaccination, and met Yamaguchi’s criteria. A PubMed literature search, performed up until March 2022, identified 10 such cases. A total of 11 cases, including the one in this report, developed AOSD-like syndrome after administration of the viral vector (ChAdOx1) vaccine (six patients) and mRNA vaccine (five patients: BNT162b2 in four and mRNA-1273 in one). There were four male and seven female patients, with their median (Q1, Q3) age and the onset of symptoms after vaccination being 36 years (29, 45) and 10 days (6, 13), respectively. Fever (100%), arthralgia/arthritis (90.9%), skin rashes (81.8%), and sore throat (81.8%) were the main clinical findings. Pericarditis (45.5%), myocarditis/cardiac dysfunction (36.4%), pleuritis (54.6%), and pulmonary infiltrations (36.4%) were also common. One patient developed macrophage activation syndrome. One patient responded well to non-steroidal anti-inflammatory drugs, and the other six showed a good response to high-dose corticosteroids alone. Of the remaining four patients, who showed partial responses to high dose corticosteroids, showed good responses to biological agents. AOSD-like syndrome following COVID-19 vaccination shared many similar clinical features and treatment outcomes to those of idiopathic AOSD (but with a higher prevalence of cardiopulmonary involvement in the former). Physicians should be aware of this extremely rare complication to achieve early diagnosis and provide proper management.

## 1. Introduction

**Figure 1:**
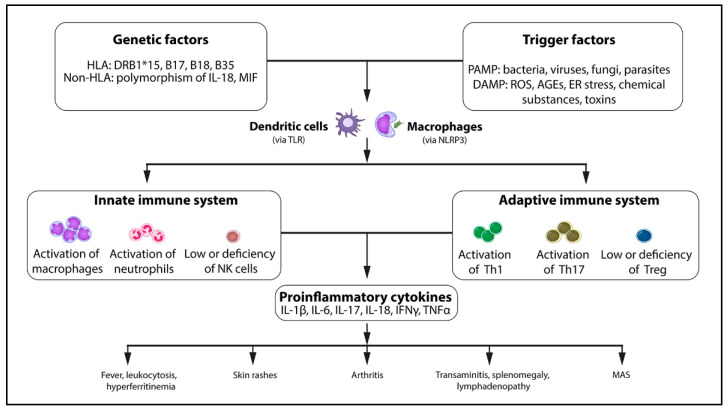
Pathogenesis of adult-onset Still’s disease (AOSD). AGEs = advanced glycation end products, DAMP = damage-associated molecular pattern, ER = endoplasmic reticulum, HLA = human leukocyte antigen, IFN = interferon, IL = interleukin, MAS = macrophage activation syndrome, MIF = macrophage inhibitory factor, NK cell = natural killer cell, NLRP3 = NOD-like receptor family pyrin domain-containing 3, PAMP = pathogen-associated molecular pattern, ROS = reactive oxygen species, Th1 = T helper 1, Th17 = T helper 17, TLR = Toll-like receptor, TNF = tumor necrosis factor, Treg = regulatory T cell.

Adult-onset Still’s disease (AOSD) is a rare inflammatory disorder that usually affects young adults. It is characterized by high spike fever, transient evanescent skin rashes, arthralgia or arthritis, sore throat, leukocytosis, and elevation of liver enzymes. Although the etiopathogenesis of this disease is not clear, evidence has shown that various mechanisms contribute to the pathogenesis, including genetic susceptibility, triggering factors, particularly infections, cytokine storm syndrome, and activation of the innate and adaptive immune system, which leads to the release of several inflammatory cytokines, thus causing inflammation in various organ systems (Figure 1) [1,2]. Diagnosing this disease is clinically challenging, as clinical manifestations can mimic infections, autoimmune diseases, and malignancies, which must be ruled out prior to diagnosis [3,4].

During the pandemic of severe acute respiratory syndrome coronavirus 2 (SARS-CoV-2), or coronavirus disease 2019 (COVID-19), people worldwide have been encouraged to perform social distancing, take precautions against infection, and receive COVID-19 vaccinations to prevent severe disease from infection. It is clear that vaccination is not without complications. A recent review by Chen et al. [5] found that various autoimmune syndromes have been reported after COVID-19 vaccination, such as vaccine-induced thrombotic thrombocytopenia (VITT), immune thrombocytopenia purpura (ITP), autoimmune liver disease, Guillain–Barré syndrome, inflammatory arthritis, Graves’s disease, and systemic lupus erythematosus. In addition, flares of pre-existing autoimmune disease after COVID-19 vaccination have also been reported [6]. Of these autoimmune syndromes, AOSD-like syndrome has rarely been described. It is interesting that a PubMed literature search, performed up until March 2022, identified seven articles (10 cases) of AOSD-like syndrome following COVID-19 vaccination [7,8,9,10,11,12,13].

This study reports a case of AOSD-like syndrome following COVID-19 vaccination, reviews the clinical manifestations and outcomes of treatment for such patients, and compares them with idiopathic AOSD.

## 2. Case Report

A previously healthy 31-year-old female with heterozygous thalassemia HbE was admitted to a local hospital after 3 weeks of prolonged high-grade fever. She had been given two doses of CoranaVac vaccine 4 and 3 months previously without adverse reactions. Symptoms of fever, together with myalgia and arthralgia, and headache had started 10 days after receiving the third viral vector (ChAdOx1) COVID-19 vaccine. She had no respiratory, gastrointestinal or genitourinary symptoms, or skin lesions. Her current symptoms persisted despite acetaminophen therapy. Physical examination showed no significant abnormalities, except for generalized arthralgia and myalgia. Initial laboratory investigations, including complete blood counts, urine analysis, electrolytes, renal and liver function tests, blood and urine cultures, chest radiography, anti-human immunodeficiency virus (anti-HIV), and serology for dengue hemorrhagic fever, scrub typhus and leptospirosis were all normal or negative, except for moderate leukocytosis (18,200 cells/mm^3^ with 87% neutrophils). An empiric antibiotic was given, but with no response. As the patient still had high fever, arthralgia, myalgia, and progressive dyspnea one week later, she was subsequently transferred to Chiang Mai University Hospital, Thailand, for further management. The patient had no history of tobacco, alcohol or herb abuse. There was no history of autoimmune diseases in her family.

The patient was alert, but obese (body mass index 34.04 kg/m^2^), and she had dyspnea and desaturation that required intubation. She was admitted to the intensive care unit. Vital signs showed a temperature of 39.9 °C, blood pressure of 107/65 mmHg, pulse rate of 130/min, and respiratory rate of 30/min. The physical examination was significant for mild pallor, tachycardia without cardiac murmurs or pericardial rubs, minimal bilateral basal lung crepitation, generalized myalgia, and arthralgia of the peripheral joints. There was no evidence of peripheral lymphadenopathy, skin rashes, oral or genital ulcers, vasculitic lesions, or hepatosplenomegaly. Due to the patient being intubated but not fully sedated, she was still alert and cooperative. Therefore, although a complete neurological examination was not performed due to limitations, the result was seemingly normal.

Significant laboratory abnormalities comprised moderate anemia (hematocrit of 27.5 vol%), leukocytosis (wbc 32,540/mm^3^ with 97.3% neutrophils), erythrocyte sedimentation rate (88 mm/h), high-sensitivity C-reactive protein (CRP) (263 mg/dL), hypoalbuminemia (2.9 gm/dL), transaminitis (AST 161 u/L and ALT 98 u/L), hyperbilirubinemia (total bilirubin 3.58 mg/dL and direct bilirubin 3.17 mg/dL), LDL (545 u/L), troponin-T (13.5 pg/mL), NT-proBNP (468 pg/mL), and ferritin (97,592 ng/mL). A chest radiograph showed mild cardiomegaly without definite infiltrates. An electrocardiogram (ECG) revealed sinus tachycardia, diffuse ST elevation with PR depression in anterolateral and inferior leads, and ST depression with PR elevation in the aVR lead, which was consistent with the pericarditis pattern. Transthoracic echocardiography showed hyperdynamic left and right ventricles without pericardial effusion or vegetation. Computed tomography of the chest and abdomen revealed minimal bilateral pleural effusions, minimal pericardial effusion, and multiple sub-centimetric lymphadenopathy with some prominent hilar nodes, but without pulmonary infiltrations or hepatosplenomegaly. Bone marrow studies showed reticuloendothelial cell hyperplasia with no evidence of hemophagocytic activity, hematologic malignancies or bone marrow invasions by bacteria or fungi.

Other investigations, including serum muscle enzymes, lipase, amylase, coagulogram, and serum complements, were all normal. Thyroid function tests were compatible with euthyroid sick syndrome. Reverse-transcriptase polymerase chain reaction (RT-PCR) for SARS-CoV-2 was negative. The infection workup, including blood and urine cultures, as well as serologies for infections, including hepatitis B and C viruses, HIV, scrub typhus, melioidosis, and leptospirosis, were negative. Immunologic studies for immune-mediated diseases, including rheumatoid factors, anti-cyclic citrullinated peptides, anti-nuclear antibodies, anti-double-stranded DNA, anti-Smith, anti-ribonucleoprotein, anti-Sjögren’s syndrome antigen A and B, anti-cardiolipin, lupus anticoagulants, and anti-β2 glycoprotein-1 antibodies, were also negative.

The broad-spectrum antibiotic meropenem was given with no response. Seven days after extensive investigations, infections or malignancies could not be identified, and AOSD was diagnosed based on Yamaguchi’s criteria (fever, arthralgia, and leukocytosis with prominent neutrophils, transaminitis, and lymphadenopathy) [14]. Intravenous dexamethasone at 20 mg/day was administered. A dramatic response occurred on the next day, with the fever resolved together with improved clinical symptoms of arthralgia, myalgia, and headache. The patient was discharged after 2 weeks of hospitalization. At the 4-week post-discharge follow-up, she was doing well and all of the laboratory parameters had returned to normal.

## 3. Review of the Literature

Details of the clinical features, laboratory findings, and treatment outcomes of the previously reported 10 cases [7,8,9,10,11,12,13] and this present one are shown in Table 1. The patient in this case report had received two doses of Sinovac followed by one of viral vector ChAdOx1 (AstraZeneca]) vaccine, which caused the onset of AOSD-like syndrome, whereas five of the other patients developed AOSD-like syndrome after the first dose of ChAdOx1 (AstraZeneca). The remaining five patients received mRNA vaccines (BNT162b2 (Pfizer) in four and mRNA-1273 (Moderna) in one) and developed AOSD-like syndrome after one and two doses of the mRNA vaccines in two and three cases, respectively. There were four male and seven female patients, with their median (Q1, Q3) age and the onset of symptoms after vaccination being 36 years (29, 45) and 10 days (6, 13), respectively. Two patients had onset at 21 and 90 days after vaccination, respectively. One patient had rheumatoid arthritis in their family and another was a thalassemia patient. RT-PCR was performed for SARS-CoV-2, as mentioned in 10 of the patients who were negative.

All of the patients met Yamaguchi’s or Fautrel’s criteria for diagnosis of AOSD (Yamaguchi’s criteria in seven, Fautrel’s in two, and both Yamaguchi’s and Fautrel’s in two). High spike fever, arthralgia, sore throat, typical skin rashes, and arthritis were present in 11, 10, 9, 9, and 4 patients, respectively. Lymphadenopathy was documented in five patients (four by imaging study) and splenomegaly in two (both by imaging study). None had hepatomegaly. Other non-criteria clinical manifestations included myalgia in six patients, chest pain in three, pericardial effusion in five, myocardial dysfunction or myocarditis in four, pleuritis or pleural effusions in six, pulmonary infiltrates in four, and pulmonary hemorrhage in one. Other uncommon manifestations included two patients with headache and hypotension, and one patient with diarrhea, hepatic failure, hyperbilirubinemia, and macrophage activation syndrome (MAS).

All of the patients had leukocytosis, with white blood counts ranging from 10,400 to 40,000 cells/mm^3^, and polymorphonuclear neutrophils of 73.0–97.3%. Bone marrow studies were performed on four patients (normal in two, compatible with MAS in one, and increased reticuloendothelial cells in one). Transaminitis and elevated lactate dehydrogenase were present in 9 out of 10 and 3 out of 3 of the patients mentioned, respectively. Rheumatoid factors and anti-nuclear antibodies were negative in all of these patients. Serum ferritin, troponin, NT-proBNP, and pro-calcitonin were increased in 10, 5, 2, and 1 out of 2 patients, respectively, of those already mentioned. Only one patient had positive autoimmune profiles (anti-β2 glycoprotein-1 and lupus anticoagulant).

One patient only responded well to non-steroidal anti-inflammatory drugs. Of the remaining 10 patients, 5 showed a good response to high-dose corticosteroids or intravenous methylprednisolone (IVMP) alone, after which the fever and other symptoms dramatically disappeared. One male patient’s fever also rapidly resolved after high-dose prednisolone (1 mg/kg), but he had an acute cardiac event 3 days later. He showed a good response after IVMP (at 1 gm/day) for 3 days. Of the remaining four patients who showed partial responses, the first one did not respond to IVMP or intravenous immunoglobulin (IVIG), but showed dramatic improvement after anakinra. Two of the others also showed dramatic improvement after receiving tocilizumab (one intravenously and the other subcutaneously). Despite the disappearance of fever after IVMP or IVIG treatment, liver function abnormalities persisted in the fourth patient, but the liver function improved dramatically after intravenous tocilizumab.

## 4. Discussion

The patient presenting with prolonged fever, arthralgia, leukocytosis, elevated liver enzymes, and lymphadenopathy without identifiable causes, including infections or malignancies, was diagnosed with AOSD-like syndrome according to Yamaguchi’s criteria. Due to the temporal relationship, this AOSD-like syndrome was more likely related to the ChAdOx1 COVID-19 vaccine than the CoranaVac vaccine. This patient responded well to high-dose corticosteroids.

When compared with recent large-sample idiopathic AOSD studies (n > 100), the clinical features and laboratory abnormalities in AOSD-like syndrome following COVID-19 vaccination were similar in general (prolonged high spike fever, skin rashes, sore throat, and arthralgia or arthritis) (Table 2) [15,16,17,18,19,20,21]. It is interesting that cardiopulmonary involvement seemed to occur commonly. A majority of the patients responded well to high-dose corticosteroid therapy, with only a small proportion of them requiring anti-cytokine therapy, which was similar to those with idiopathic AOSD. Whether the natural course of this syndrome was also similar to those with idiopathic AOSD is unknown, due to the short follow-up duration of the reported cases.

Whether the high prevalence of cardiac involvement in AOSD-like syndrome following COVID-19 vaccination was related to cardiac complications from either the viral vector ChAdOx1 or mRNA vaccine is not known. A study from the United Kingdom showed an increased risk of myocarditis ranging from 1 to 10 events per 1 million people vaccinated. The risk was associated with the first dose of ChAdOx1 and BNT162b2 vaccines, and the first and second doses of the mRNA-1273 vaccine, which usually developed over a 1- to 28-day post-vaccination period. In addition, the subgroup analysis by age showed that the risk was associated with the second dose of mRNA-type vaccine only in those who were under 40 years old. The prevalence of pericarditis was less common than that of myocarditis [22]. These rates are much lower than the prevalence rate of viral myocarditis (10 to 22 per 100,000 individuals) [23]. Six myocarditis cases in young adults and children were reported to have received an mRNA-based vaccine, and it was found that myocarditis was common in males with no abnormal ECG findings, except for atrial tachycardia. No significant abnormalities were observed on echocardiography, except for one female patient, who had pericardial effusion. Myocarditis was diagnosed in all of the patients on cardiac magnetic resonance imaging, which showed myocardial edema and late gadolinium enhancement in the lateral wall of the left ventricle, without impaired cardiac contractility. The pericarditis and myocarditis patients responded well to colchicine and ibuprofen [24]. The prevalence of pericarditis and pericardial effusion, together with other clinical manifestations, was seen to be higher in the AOSD-like patients in this study, shortly after COVID-19 vaccination, which favored cardiac involvement, due, in part, to the clinical manifestations of AOSD-like syndrome, rather than the cardiac complications of COVID-19 vaccines. However, it might not be possible to observe a clear-cut differentiation between these two conditions [25], as the possibility of coincidence remains.

The pathogenesis of AOSD-like syndrome following COVID-19 vaccination has not been understood clearly. Molecular mimicry and bystander activation are two possible mechanisms that have been postulated in the development of autoimmune disease after vaccination, particularly in those who have susceptible major histocompatibility complex (MHC) alleles [26,27,28]. Microbial antigens or peptides, as components of vaccines, presented by MHC class II, can stimulate both innate and adaptive immune cells, causing the release of pro-inflammatory cytokines (Figure 1). The nucleic acids in both the viral vector and mRNA vaccines can act as pathogen-associated molecular patterns (PAMPs) and activate Toll-like receptors [29,30], which subsequently stimulate RNA sensors, causing maturation of the dendritic cells, stimulation of MHC molecule expression, and cytokine production [29,30]. The spike proteins of COVID-19 have shown the ability to stimulate both innate and adaptive immunity, causing overproduction of cytokines observed approximately 2 weeks after vaccination [31,32,33,34]. The clinical features of high fever, multisystem involvement, very high ferritin levels, and rapid response to high-dose corticosteroids or anti-cytokine therapy suggested that cytokines might play an important role in the pathogenesis of this condition, which resembles that observed in idiopathic AOSD [3,4]. Unfortunately, cytokine levels have rarely been determined in AOSD-like syndrome following COVID-19 vaccination, except for a high level of IL-6 and IL-2R in one patient after the ChAdOx1 vaccine, reported by Leone et al. [7]. In addition, a genetic study was undertaken in only one patient with AOSD-like syndrome after BNT162b2 vaccination [12]. Five heterozygous genetic variants of uncertain significance were identified, with no specific mutations of autoinflammatory diseases, hemophagocytic lymphohistiocytosis, or primary immune deficiency.

Not only rheumatic syndromes after COVID-19 vaccination, but also new onset across a wide spectrum or flares from pre-existing rheumatic and autoimmune diseases after COVID-19 infection have been reported and reviewed [35,36,37,38]. Several reviews have shown that COVID-19 can stimulate both innate and adaptive immunity [39,40]. 

The proposed mechanisms by which COVID-19 induces autoimmune diseases have included molecular mimicry, bystander activation, superantigenicity, type I interferon stimulation, and breaking of tolerance, which are similar to those seen in other viruses [39,40,41].

Considering the number of COVID-19 vaccines administered—currently 1 billion doses worldwide—the 11 reported AOSD-like syndrome cases that followed them would be considered extremely rare. Whether this syndrome is an extremely rare adverse reaction to the vaccine or coincidental is not known. Sporadic cases of AOSD flares after ChAdOx1 vaccine [42,43] and the development of MAS after BNT162b2 vaccine [44] administration have been described. Another report found that the incidence of adverse events was similar between patients with rheumatic diseases and healthy controls, with a low incidence of flare (<5%) among the former [45]. A recent meta-analysis on the effect of COVID-19 vaccine booster doses in patients with pre-existing immune-mediated rheumatic diseases found that the incidence of local and systemic adverse events, including disease flares, was either comparable or slightly increased when compared with the primary series of vaccinations [46]. This information suggests a very low risk of vaccines that might trigger a flare. Nevertheless, the risk of communicable COVID-19 infection is still high and can cause more serious conditions. Therefore, in those developing AOSD-like syndromes after COVID-19 vaccinations, the risk and benefit from receiving additional or booster doses of the COVID-19 vaccine could be discussed thoroughly between physicians and patients.

## 5. Conclusions

The clinical features and treatment outcomes of AOSD-like syndrome following COVID-19 vaccination were similar to those seen in idiopathic AOSD, but with rather common cardiopulmonary involvement in the former. As COVID-19 vaccination is being administered increasingly worldwide, more cases of AOSD-like syndrome following vaccination, although extremely rare, are expected to be seen. Physicians should be aware of this condition in patients who have prolonged fever shortly after COVID-19 vaccination, and diagnosis should be prompt for proper early management, in order to avoid potential serious complications.

## Figures and Tables

**Table 1:** Characteristics of adult-onset Still’s disease-like syndrome associated with COVID-19 vaccination.

Authors. [Ref], Year	Leone F. [7], 2021	Magliulo D. [8], 2021	Sharabi A. [9], 2021		Park SY. [10], 2021	AlQudari EA. [11], 2022	Baicus C. [12], 2021	Padiyar S. [13], 2022			Present Case, 2022
No. of cases	1	1	1	2	1	1	1	1	2	3	1
Type of vaccine	ChAdOx1	mRNA-1273	BNT162b2	BNT162b2	BNT162b2	ChAdOx1	BNT162b2	ChAdOx1	ChAdOx1	ChAdOx1	ChAdOx1
Age in years	36	45	43	56	36	29	22	20	47	35	31
Sex	M	F	M	F	F	M	M	F	F	F	F
Underlying disease	N	N	N	N	N	N	N	N	N	N	Thalassemia
Family history of autoimmune disease	N					N			Y (RA in elder sister)		No
Dose of vaccination	1st	2nd	2nd	2nd	1st	1st	1st	1st	1st	1st	1st
Onset of symptoms after vaccination (days)	6	5	10	7	10	2	13	10	21	90	10
RT-PCR for SARS-CoV-2	Neg.	Neg.	Neg.		Neg.	Neg.	Neg.	Neg.	Neg.	Neg.	Neg.
**Diagnostic criteria**	Yamaguchi	Yamaguchi	Yamaguchi	Yamaguchi	Yamaguchi	Yamaguchi	Yamaguchi and Fautrel	Fautrel	Fautrel	Yamaguchi and Fautrel	Yamaguchi
**Clinical features**											
Fever > 39 °C	Y	Y	Y	Y	Y	Y	Y	Y	Y	Y	Y
Arthralgia	N	Y	Y	Y	Y	Y	Y	Y	Y	Y	Y
Arthritis	N	N	Y	Y	N	Y	N	N	Y	N	N
Typical skin rashes	Y	Y	Y	Y	Y	Y	Y	Y	N	Y	N
Leukocytosis (PMN %)	30,380 (86.6%)	22,100 (88.7%)	12,500 (>95%)	40,000 (>80%)	12,220 (NS)	26,200 (87.4%)	Y (number and % NS)	10,400 (73.0%)	12,100 (82.0%)	11,700 (75.0%)	32,540 (97.3%)
Sore throat	Y	Y	Y	Y	Y	Y	Y	Y	N	Y	N
Lymphadenopathy	Y (CT)	Y (CT)	N	N	Y (CT)	N	N	N	N	Y	Y (CT)
Hepatomegaly or splenomegaly	Splenomegaly (CT)				Splenomegaly (CT)	N		N	N	N	N
Elevated liver enzymes	Y	Y	N	Y		Y	Y	Y	Y	Y	Y
RF and ANA	Neg. (NS)	Neg.	Neg.	Neg.	Neg.	Neg.	Neg. (NS)	Neg.	Neg.	Neg.	Neg.
LDH, u/L			High							>1000	545
**Other clinical manifestations**	Chest pain, pericardial effusion (echo), bilateral pleural effusion (CT), myopericarditis (PET-CT)	Myalgia, pleurisy, bilateral pulmonary infiltrates, hypoxic respiratory failure	Myalgia, dyspnea, bilateral pulmonary infiltrates and effusions (CT), hypoxemia, regional left ventricular dysfunction (echo)	Chest pain, shortness of breath, weakness, pericardial effusion (echo), bilateral crepitation on both lungs and pleural effusion	Dyspnea, pericardial effusion (CT), and bilateral pleural effusions (CT)	Pulmonary infiltrates (CT), hypotension, hypoxemia	Myalgia, chest pain, ST elevation (ECG), LV hypokinesia (echo), myocarditis (MRI), diarrhea, hypotension	Myalgia	Weight loss, loss of appetite, pericardial effusion (echo)	Weight loss, pulmonary hemorrhage, hepatic failure, MAS	Dyspnea, hypoxemia, myalgia, headache, pericardial effusion and bilateral pleural effusion (CT), cardiomegaly and hyperdynamic heart (echo), abnormal ECG, hyperbilirubinemia
Bone marrow study								normal	normal	MAS	Increase RE activity
Ferritin, ng/mL	1482	2911		49,149	4712	>2000	54,921	11,491	404	>100,000	97,592
Troponin, pg/mL	1695	350	52	535			high				13.5
NT-proBNP, pg/mL		1815									468
Procalcitonin, ng/mL							33			normal	
Autoimmune panels	β2GP1+, LA1+	CCP-	ENA-, MPO-, PR3-	CCP-, ANCA-				CCP-		CCP-, autoimmune study negative	CCP-, dsDNA-, Sm-, RNP-, SSA-, SSB-, ACL-, LA1-, β2GP1-
**Initial treatment**	MP 0.75 mg/kg	Pred. 1 mg/kg	IVMP 1 gm/day ×3 days	Pred. 1 mg/kg	IVMP 1 gm ×3 days	Pred. 1 mg/kg	IV Dexa. 16 mg/day	Naproxen 500 mg/day	Dexa. 16 mg/day	IV-MP 1 mg/×3 days plus IVIG 2 gm/kg ×5 days	IV Dexa. 20 mg/day
**Outcomes**	Rapid response	Rapid response	Rapid response	Rapid response	Partial response. Dramatic response to IV tocilizumab	Rapid resolution of fever in 2 days, but developed cardiovascular event requiring intubation. Marked improvement after IVMP 1 gm/day ×3 days followed by pred. 100 mg/day.	Partial response. No improvement after IVMP or IVIG. Good response to anakinra	Rapid response	Partial response. Marked improvement after SC tocilizumab	Rapid resolution of symptoms, but not liver functions. Marked improvement of liver functions after IV tocilizumab	Rapid response

Blank = not mentioned or not available. NS = not specified, Y = yes, N = no, Neg. = negative, - = negative, + = positive. ACL = anti-cardiolipin antibodies, ANA = antinuclear antibodies, ANCA = anti-neutrophilic cytoplasmic antibodies, CCP = anti-cyclic citrullinated peptide antibodies, CT = computed tomography, Dexa. = dexamethasone, dsDNA = anti-double-stranded antibodies, ECG = electrocardiogram, Echo = echocardiogram, ENA = anti-extractable nuclear antigen antibodies, IV = intravenous, IVIG = intravenous immunoglobulin, IVMP = intravenous methylprednisolone, LA1 = lupus anticoagulants, LDH = lactate dehydrogenase, LV = left ventricle, MAS = macrophage activation syndrome, MP = methylprednisolone, MPO = anti-myeloperoxidase antibodies, PET-CT = positron emission tomography—computed tomography, PMN = polymorphonuclear cells, PR3 = anti-proteinase-3 antibodies, Pred. = prednisolone, RE = reticuloendothelial, RF = rheumatoid factors, RNP = anti-ribonucleoprotein antibodies, Sm = anti-Smith antibodies, SSA = anti-Sjögren’s syndrome A antibodies, SSB = anti-Sjögren’s syndrome B antibodies, β2GP1 = anti-β2 glycoprotein-1 antibodies.

**Table 2:** Clinical features of adult-onset Still’s disease (AOSD)-like syndrome following COVID-19 vaccination in comparison with idiopathic AOSD (selected series).

Authors. [Ref], Year	AOSD-like Syndrome Following COVID-19 Vaccination	Kalyoncu U. [15], 2016	Sfriso P. [16], 2016	Hu QY. [17], 2019	Nakamura H. [18], 2020	Li R. [19], 2021	Sugiyama T. [20], 2022	Ruscitti P. [21], 2022
Country		Turkey	Italy	China	Japan	China	Japan	Italy
Type of study	Cases review	Retro., multicenter	Retro., multicenter	Retro., multicenter	Retro., multicenter	Retro., single center	Retro., multicenter	Retro., multicenter
Number of patients	11	356	245	517	178	492	216	194
Female, %	63.6	59.0	47.3	72.0	70.2	78	75.9	47.4
Age at onset, years, Median (Q1, Q3)	36 (29, 45)	30	38.8	37.7	42	37.1 ± 14.3 (mean ± SD)	51.6	41
**Clinical characteristics**								
Fever, %	100.0	95.8	92.6	91.3	96.1	98.6	99.5	98.5
Arthralgia, %	90.9	94.9	93	73.1	73.6	76.8	79.4	83.5
Arthritis, %	36.4	64.6	75.8					59.8
Skin rashes, %	81.8	66.9	67.7	79.9	62.9	84.8	90.7	73.2
Sore throat, %	81.8	63.7	61.8	60.5	39.3	63.0	63.3	59.3
Lymphadenopathy, %	45.5	28.1	60.4 ^a^	51.1	37.1	51.0	63.5	52.1
Hepatomegaly, %	0	25.0	41.7	6.6	36.5 ^b^	6.7	NA	56.7 ^d^
Splenomegaly, %	18.2	25.0	60.4 ^a^	34.4		28.5	49.5	45.9
Pericarditis/pericardial effusion, %	45.5	6.2	17.3	14.1	10 ^c^	5.9	7.4	20.6
Myocarditis/cardiac dysfunction, %	36.4	NA	NA	NA	NA	NA	0	NA
Pleuritis/pleural effusions, %	54.5	7.9	NA	23.9	10 ^c^	NA	12.5	19.1
Pulmonary parenchyma involvement, %	36.4	NA	NA	NA	NA	15.4	2.3	9.3
Myalgia, %	54.5	52.8	NA	32.5	NA	25.2	NA	60.3
MAS	9.1	2.5	2.9	NA	10.7	6.7	22.3	11.9
Leukocytosis, %	100.0	84.9	81.0	85.6	55.1	NA	NA	62.9 (>15,000)
Elevated liver enzymes (AST/ALT), %	9/10 (90.0) ^e^	50.4/47.9	53.5	61.6	53.9	NA	81	56.7 ^d^
Negative RF, %	11	99.1	96.2	94.0	NA	NA	NA	NA
Negative ANA, %	11	98	90.4	90.8	NA	NA	NA	NA

Retro. = retrospective study, MAS = macrophage activation syndrome, AST = aspartate aminotransferase, ALT = alanine aminotransferase, NA = not available, RF = rheumatoid factors, ANA = anti-nuclear antibodies. ^a^ = lymphadenopathy/splenomegaly, ^b^ = hepatosplenomegaly, ^c^ = serositis, ^d^ = liver involvement, ^e^ = 9 out of 10 for whom the data were available.

## Data Availability

Not applicable.
